# Meeting of the American Medical Association

**Published:** 1855-09

**Authors:** 


### EIGHTH ANNUAL MEETING OF THE AMERICAN MEDICAL ASSOCIATION.

(CONCLUDED.)

Dr. Sanford B. Hunt resumed the reading of his report on the hygrometrical state of the atmosphere, in various localities. Pending the reading of the account, a motion was made and adopted, "that the desultory conversation of members be dispensed with." The report abounds with interesting facts on the subject of epidemical diseases, during the summer of 1854.

It was accepted, and referred to the committee on publication.

A resolution was adopted, providing that a committee be appointed to confer with the directors of the several railroad and steamboat companies, with the view of having commutation tickets issued to the delegates to the convention, to be held at Detroit next year.

Dr. Frank H. Hamilton, of Buffalo, N. Y., then submitted a report on "Deformities after Fractures." The report was not complete, the author having only considered fractures of the ossa nasi, septum nasi, superior maxilla, inferior maxilla and clavicle. Copious statistics accompanied each fracture.

Dr. Hamilton said he had a word to say which did not belong to the report. Prosecutions for malpractice have become so frequent that surgeons were alarmed, and not a few were abandoning the profession, or refusing altogether to undertake the treatment of grave surgical accidents, and especially of fractures. So frequent were these prosecutions that members were no longer surprised at such statements. If they had heard the speaker say that lawyers were abandoning their profession from this cause, they would have been startled, but to us the fact is familiar.

It is proper for us, then, to interrogate ourselves. Why is it that we are held to an accountability so much more strict than any other professional men, or than any other artizans? Is it because there are jealous and designing men in our own ranks who instigate these suits? No doubt such men may be found, but only as an exception. The fact is that surgeons have sometimes been mulcted in damages simply because the jury believed, from the united character of the medical testimony, that it was a conspiracy, and the more conclusive the testimony, the more certain, with some jurors, is the defendant to suffer.

Is it chargeable to the members of another profession—to the lawyers? There may be some men in the profession of law, also, who, driven by the sheer necessity of their circumstances—by their extreme poverty, or who, without any such apology, with only loose notions of right and wrong, encourage and undertake such suits—such are the men who hang about the tombs in New York, and who may be found, more or less, in every town—but the speaker has reason to believe that honorable and intelligent lawyers seldom countenance these prosecutions. That eminent jurist of the state of New York, Joshua Spencer, has told Dr. Hamilton, that for himself he does not think he ever commenced a suit of this character, although he has been frequently retained as counsel, and he believes his brethren, generally, look upon these complaints with suspicion and refuse to meddle with them.

Where then, must we look for an answer to the question, Why are these prosecutions against surgeons so frequent? Let the gentleman be assured, the causes are to be found in the very imperfections of our art, and in our own unwillingness to admit these imperfections. Surgeons have claimed too much, and it cannot certainly be expected that the world will demand of them less than they claim for themselves. Again and again surgeons have said that a fracture of the femur might be generally made to unite without any shortening, while the fact is not so. Malgaigne, who is eminently an honest man, says, to make this bone unite in an adult person, where the fracture is sufficiently oblique to prevent the ends from supporting each other, is "simply impossible" (*simplement impossible.*)

Let the profession be wiser in future and acknowledge that they cannot perform impossibilities.

Dr. Charles Hooker, of New Haven, read a report upon the "Diet for the Sick." The document lays down certain laws for the government of diet in the various diseases "flesh is heir to," and specific articles that may be given to patients. Referred to the committee on publication.

A resolution expressing the thanks of the association to the ex-President and other officers, for the ability with which they have discharged their duties, was adopted.

At this period the association adjourned to proceed to Independence Hall. On arriving at this place, the delegates were introduced to Mayor Conrad, by Dr. Isaac Hays, chairman of the committee of arrangements.

Dr. Hays briefly addressed his Honor the Mayor, giving an outline of the history of the association, and alluding to the patriotism of the profession as exemplified in Rush, Warren, and others, closed as follows:

"But I may assure you that the spirit which animated our ancestors, if it appear dormant, is not extinct in our bosoms, and that while standing on this spot—the shrine sacred to Human Liberty,—we experience feelings akin to those which the inspired Lawgiver must have felt when he heard the voice calling to him out of the midst of the burning bush—"Put off thy shoes from off thy feet, for the place whereon thou standest is holy ground." (Loud Applause.)

The truly eloquent reply of Mayor Conrad will well repay perusal.

REPLY OF MAYOR CONRAD.

"Mr. Chairman of the Committee of Arrangement, I thank you in the name of the community which I have the honor to represent, for your eloquent introduction of our friends to the authorities of the city, and to this, the Hall of Independence.

Gentlemen of the American Medical Association, I am proud of the privilege of extending to you, in the name of the government, and of the people, of Philadelphia, a most cordial welcome. (Applause.)

I bid you welcome to our city—a city which, deriving a cherished distinction from the profession which you adorn, is eager, now and ever, to requite it in her tribute of respect for its professors.—I welcome you to our people, whose intercourse, for many a year, with you or your brethren, has inspired a feeling which, reserved as we are sometimes said to be, will, I doubt not, burst into earnest and unambiguous expression, before you leave us. (Applause.)

I welcome you, gentlemen, to this Hall, but not as strangers, or the sons of strangers—for it is your own. As the temple and territory of Delphos, in the wildest domestic perturbations of Greece, afforded one sacred area over which the cloud of discord never gathered, one altar whose worship was never invaded, this spot, consecrated to our common American glory, knows no lines of latitude, and belongs, in truth, no more to us, whose peculiar privilege it is to inherit its guardianship, than to our brothers—to you. In coming hither, therefore, you come home. (Applause.)—These precincts have been hallowed, for all time, by the heroic virtues of your and our fathers. This is the fountain from the which the living waters of American liberty were first drawn, and it is, therefore, most sacred—(woe to the generation in which it ceases to be sacred!)—but, like the well of the of the Patriarch, all the tribes of Liberty's Israel own here an equal right, and owe here an equal homage. (Great Applause.)

In no sense, then, can I greet you as strangers, for yours are names familiar to every American proud of the science of his country, and those who are united, by this association, in a cause so lofty as that eloquently characterized by your chairman, may not only claim the universal and acknowledged privileges of the republic of minds, but the rights of a nearer and dearer charter, the brotherhood of beneficence—the kindred claims of noble hearts, knit in the highest and holiest of human aspirations. In this spirit, with the most fervent and fraternal sentiments of respect and regard, I greet and welcome you.

You are right, Mr. Chairman, in claiming, amid the associations which hallow these precincts, a peculiar privilege for your profession—a profession which not only sprinkled, with the first blood of the revolution, the highest altar upon which valor vowed and dedicated our country to freedom—I refer, as you have referred, to Dr. Warren and Bunker Hill—but which, in every struggle for the enlargement and enlightenment of human destinies, has been eminently distinguished for courage, zeal and fidelity to the rights of man. You have, therefore, a peculiar right to claim kindred here, and have that claim allowed; and within these walls which witnessed the zeal of Rush, it would be treason to virtue to forget that one of the lights of your profession shed glory upon the solemn debates of this hall, and was foremost among those that bade yonder bell, (preserved and devoted to the veneration of posterity,) with its iron tongue to "*proclaim liberty throughout all the land to all the inhabitants thereof.*" (Continued applause.)

It is the glorious peculiarity of your profession that, while ambition, in its ordinary and most applauded paths, plays the part of *the Destroyer*, and wins glory at the expense of human life and happiness, you and yours, with a more exalted civilization and a nobler heroism, have ever *sought to save.* Next to the highest of all human courage—if, indeed, it be merely *human*—that of the martyrs of religious truth, the courage of the physician, whether on the battle-field or in the lazar-house, the courage of science and humanity, is the most sublime, and best entitled to the *clarum et venerabile nomen.* The vulgar courage of the warrior, under the base stimulus of passion or the low greed of applause, can hardly be compared to the noble intrepidity of the surgeon, who gleans, in the ruthless and red-handed reaper's path, the leavings of battle; and still less with the hero of the hospital, who encounters the grim antagonists in the horrid silence and gloom of the pestilence. Imagination can hardly embody an instance of human courage and virtue more sublime and unearthly than that of the physician, who in the midnight of a plague-stricken city, midst the fœtid solitudes of its alleys, and entering the devoted hovel of the wretched, ministers—while only pestilence, and misery, and death, and God look on, to the perishing. I need not step from this spot to grasp the hand of many a hero who claims no laurel—many a noble philanthropist whose sacred labors in scenes like these, have been unmarked, save by the eye that never slumbers, and remembered only by Him, who alone can reward.

To such a profession, one venerable from its antiquity, noble from the grandeur of its objects, illustrious from its achievements, and which demands every aid and energy of genius and science, of head and heart that dignifies the race, it is not strange that, go where it may, a ready homage greets and a ready blessing attends it. In our own city, all that is noble in patriotism, all that is exalted in science, all that is bright and beautiful in the arts that refine society, all that is lovely and cherished and holy in private life, combine to render the profession sacred and dear to us.

There are few living to whom some one death in the past is not the sole event and solitary memory of the survivor's life—to him a lonely pyramid in the melancholy desert; and to such a mind and memory, the debt of the death-bed, where science, rendered holy by its office, ministered, though never paid is never repudiated. I never knew a good man, still less a good woman, who had not such a debt—a debt which bankrupt gratitude cherished with its holiest affections and devoutest memories.

In these times, when the omnipotence of associated effort is invoked for so much that is of dubious merit, it is a gratifying spectacle to behold the enlightened professors of the most exalted of all arts—men sage and grave, unselfish and unaspiring, forsaking the homes to which they are bound by the affections and the afflictions of thousands, by wealth and fame and influence, to wander wearily away upon a pilgrimage of hundreds of leagues, in the cause and interests of the human family, its security, its health and happiness. For more than ten years, the representatives of your profession have gathered in convention. What other body of our citizens have made a like effort—a like sacrifice? Selected from the most eminent of the profession, the delegates have been men whose years, like their virtues, were many. How difficult must have been to them the effort to burst through the bonds of a relying and clinging practice! How great the labor and how heavy the sacrifice! They have already visited in this duty, the cities of every section of our wide country. How many have fallen by the wayside? How many martyrs could you not thus number in this cause? How many of the good and great of the profession have, in these benevolent pilgrimages, joined the ranks of the thousands who have sacrificed themselves, at the requisitions of duty, as recognized and enforced by your self-imposed laws—joining the dead in the effort to aid the living. The epitaph of the Spartans at Thermopylæ, might well commemorate the virtues and the fate of these martyrs. But if the cost has been great, the results have been commensurate. 7

Of the professional advantages attained, though I know them to be invaluable, I will not presume to speak; but I may be permitted to state, as health is the most important subject of municipal provision and care, that the transactions of the association, which I have examined with great interest, comprise much that merits the attention, and will reward the respectful consideration of the municipal government of the Union.

It is natural that Philadelphia should feel, as she does feel, a profound interest in the cause of medical education in this country. She cannot, of course, forget that it was here that the first medical college was established in this country; that its merits and success extorted a reluctant trans-atlantic tribute of admiration, and that progressing rapidly, but wisely, it achieved and maintained an equality with the most celebrated institutions of the old world. As the cause of medical education has expanded, and instructions worthy of the cause and country have sprung up, each triumph, thus attained has been regarded here as the successful outbursting of an offshoot from the primary effort; and Philadelphia, while rejoicing in the expansion and elevation of medical education throughout the land, has almost fancied—so earnest is her interest in medical education—that she had a right to indulge a parental pride in all that advances that interest.

These genial feelings have been maintained, in all their early and fervid freshness, by constant intercourse with all sections of our country. The ingenuous and gallant youths that have come hither for medical instruction have, in their unstudied intercourse, exhibited the character of their respective states in a light so generous and exalted as to win our affections not only for themselves, but for the communities and states which could exult in them as their own. Winter after winter, we have had hundreds of these noble young spirits among us here. And let me remark, that rigorous as I am said to be in the administration of the law, I have yet to know the first occasion to rebuke, much less punish, a medical student. We have found them as gentle and decorous in their deportment as they are exalted in their aspirations; and had Philadelphia—eminently catholic in her affections for her sister communities—needed a lesson of love and loyalty, these young missionaries would have taught it. This interchange of sympathies has endured for the third of a century, (may it last forever!)—Her youths who formerly bore those sentiments to the remote sections of our republic, stand before me now as the revered sages and ornaments of their profession, meeting here the evidences of a reputation which had preceded them, and has long been cherished by us. And who can tell what has been the results of this kindly interchange of kindly feeling? It has doubtless been felt in every community, social, and political relation of life, correcting the prejudices, harmonizing the discords, and subduing the dangers of our common country.

We realize these facts. We recognize in the members of an enlightened profession like yours, so many patriots and philanthropists engaged in the great and general interests of the human race, and, apart from the more scientific acquisitions of your annual meetings, we perceive in them, results auspicious to all that we cherish, all that is kindly, forbearing and conservative between man and man, party and party, state and state, section and section; and so regarding them we hail and greet you with a welcome as sincere and cordial as the heart can forge or the tongue can utter. (Loud applause.)

On re-assembling at the Musical Fund Hall, it was resolved to visit the High School at 12 o'clock on Thursday, (to-day.)

On motion, the thanks of the association were tendered to Mayor Conrad, for the very cordial manner in which he had received the delegates in Independence Hall, and that a copy of his speech be printed in connection with the proceedings of this association.

Dr. Thompson, of Deleware, offered a preamble and resolutions providing for the appointment of a committee from each state to report on medical topography and epidemics. Ordered to lie over till next day.

Dr. Isaac Wood, the Treasurer, made a report, from which it appears that during the year the sum of $3216 30½ was received, of which there is a balance remaining amounting to $1115 26.

Dr. Francis Condie, from the committee on publication, made a report in refetence to certain charges made against the committee and Dr. Meigs, which gave rise to considerable discussion.

Dr. Stewart offered a resolution, expressing thanks for the manner in which the committee on publication had performed their arduous labors. Pending the discussion, the resolution and the report were both withdrawn.

Dr. Watson offered a resolution appropriating the sum of $1000 to defray the expenses of the committee to have prepared the stone for the National Monument. Adopted.

Dr. Mauran called up a preamble and resolution expressive of thanks to those United States Senators who so earnestly and ably advocated the passage of the bill relative to sickness on shipboard, as suggested by this Association.

The resolution was adopted, and copies ordered to be sent to the Senators.

Dr. Francis West, Secretary, read a paper from Dr. William H. Byford, of Evansville, Ind., on the "Pathology and Treatment of Scrofula," which, on motion, was referred to the committee on publication.

Dr. N. S. Davis, of Chicago, Ill., submitted a report on the nutritive qualities of milk, and the influence produced thereon by pregnancy and mentruations, in the human female, and pregnancy in the cow; and also on the question whether there is not some mode by which the primitive constituents of milk can be preserved in their purity and sweetness, and furnished to the inhabitants of cities in such quantities as to supercede the present defective and often unwholesome modes of supply.

The report was referred to the committee on publication.

The hour of adjournment now arrived.

At 4 o'clock yesterday afternoon, the delegates, accompanied by many ladies, visited Girard College.

3d and 4th day's proceedings next number.—*Buffalo Medical Journal.*

